# Photochemical efficiency correlated with candidate gene expression promote coffee drought tolerance

**DOI:** 10.1038/s41598-021-86689-y

**Published:** 2021-04-01

**Authors:** Meline de Oliveira Santos, Larissa Sousa Coelho, Gladyston Rodrigues Carvalho, Cesar Elias Botelho, Luana Ferreira Torres, Diego Júnior Martins Vilela, Alan Carvalho Andrade, Vânia Aparecida Silva

**Affiliations:** 1 BDTII FAPEMIG/EPAMIG Sul, Campus Universitário UFLA, Lavras, Minas Gerais Brazil; 2 Universidade Federal de Lavras, Campus Universitário, Lavras, Minas Gerais Brazil; 3 Empresa de Pesquisa Agropecuária de Minas Gerais, Epamig Sul, Campus da Universidade Federal Lavras – UFLA, Rodovia Lavras/Ijaci Km 02, Cx. P. 176, Lavras, Minas Gerais Brazil; 4 Embrapa Café, Inova Café, Campus Universitário da Universidade Federal de Lavras, Lavras, Minas Gerais Brazil; 5 Empresa de Pesquisa Agropecuária de Minas Gerais, Epamig Oeste, Patrocínio, Minas Gerais Brazil

**Keywords:** Drought, Plant molecular biology, Plant physiology, Plant stress responses

## Abstract

The aim of this study was to identify the correlation between photochemical efficiency and candidate genes expression to elucidate the drought tolerance mechanisms in coffee progenies (Icatu Vermelho IAC 3851-2 × Catimor UFV 1602-215) previously identified as tolerant in field conditions. Four progenies (2, 5, 12 and 15) were evaluated under water-deficit conditions (water deficit imposed 8 months after transplanting seedlings to the pots) and under irrigated system. Evaluations of physiological parameters and expression of candidate genes for drought tolerance were performed. Progeny 5 showed capacity to maintain water potential, which contributed to lower qP variation between irrigated and deficit conditions. However, the increases of qN and NPQ in response to stress indicate that this progeny is photochemically responsive to small variations of Ψam protecting the photosystem and maintaining qP. Data obtained for progeny 12 indicated a lower water status maintenance capacity, but with increased qN and NPQ providing maintenance of the ɸPSII and ETR parameters. A PCA analysis revealed that the genes coding regulatory proteins, ABA-synthesis, cellular protectors, isoforms of ascorbate peroxidase clearly displayed a major response to drought stress and discriminated the progenies 5 and 12 which showed a better photochemical response. The genes *CaMYB1, CaERF017, CaEDR2, CaNCED, CaAPX1, CaAPX5, CaGolS3, CaDHN1* and *CaPYL8a* were up-regulated in the arabica coffee progenies with greater photochemical efficiency under deficit and therefore contributing to efficiency of the photosynthesis in drought tolerant progenies.

## Introduction

Coffee is a tropical tree species being one of the most traded commodities worldwide. Coffee production represents an extremely important economic factor for both consumer countries and producers, where it represents the basis of income for a large number of small and medium farmers. *Coffea arabica* L. and *Coffea canephora* P. are the two species commercially relevant with approximately 60% and 40% of global production, respectively^1^. Climate change is expected to have a negative impact on production, with a consequent economic and social impact in countries. In Brazil, the largest coffee producer, drought represents a major constraint for coffee production^2^. Variations in the climate characterized by episodes of drought or irregular rainfall distribution have been observed frequently in recent years, especially those that occurred during the year 2014 e 2020 which led to the occurrence of water deficit culminating in a reduction in the harvest^3–5^.

Therefore, studies like the present, which aim to study the response to abiotic stresses such as drought, and to identify potential tolerance markers, can provide producers with fundamental tools to minimize the effects of environmental limitations. The genetic improvement program of Minas Gerais, the world’s largest coffee producer, works by associating field data with data obtained in a greenhouse to select genotypes with higher productivity coupled with physiological phenotyping to obtain high performance genotypes with multiple characteristics of interest. Tolerance to drought in the field is defined based on productivity under water deficit conditions^6^. And with that in order to carry out the physiological analyses, we started from selected genotypes under field conditions, that is, with the desired phenotype, and then carried out a more detailed phenotyping in the greenhouse in order to identify the differential mechanisms that lead to such a tolerance.

Both, *C. arabica* and *C. canephora* genotypes display variability to drought tolerance. In general, *C. canephora* tolerates prolonged periods of drought than *C. arabica*^7^. Considering that introgressed arabica progenies with *C. canephora* may have a potential drought tolerance phenotype, in this work, the focus was the study of progenies derived from crossing Icatu Vermelho IAC 3851-2 and Catimor UFV 1602-215. The Icatu germplasm was obtained from an interspecific hybridization between a tetraploid coffee plant of *C. canephora* and a plant of the cultivar Bourbon Vermelho of *C. arabica*. The catimor originated from the crossing of Caturra Vermelho with the Timor hybrid coffee trees, which in turn originated from the natural hybridization between *C. arabica* and *C. canephora*. These progenies stood out due to rust resistance, good productivity and cup quality^8^.

In general, coffee trees display a differential reduction on leaf water potential^9^ and stomatal closure in response to abscisic acid biosynthesis and signaling^10^. As a consequence, there is a reduction in the intercellular concentration of CO_2_ and a greater resistance to the mesophyll, leading to a decreasing photosynthesis rate^11^. In Brazilian coffee-growing regions, such as the south of Minas, periods of drought are commonly associated with high light intensities and temperatures^4^. Drought effects are enhanced when combined with high light intensities that lead to oxidative damage induced by an imbalance in the reaction center^12^. The light energy absorbed by the chloroplasts is dissipated via photosynthetic electron transport, chlorophyll fluorescence and heat dissipation^13^. Therefore, studies related to the chlorophyll *a* fluorescence-characteristics curves performed in response to photosynthetic-photon flux may indicate the response pattern of photosynthesis and PSII performance of progenies under water deficit^12,14–16^. In addition, these curves can not only access the photosynthetic capacity of the plant, but the potential tolerance under a large scale of radiation intensity^17^. Thus, the exploration of the physiological parameters (leaf-water potential and photosynthetic rates) under drought has helped us to identify progenies tolerant to water deficit.

At the molecular level, previous reports have identified candidate genes in different tissues (leaves, roots, plagiotropic buds) of drought-tolerant (DT) and drought-susceptible (DS) plants of *C. canephora*^18–22^ and *C. arabica*^19,23–25^ displaying differential expression profiles under drought stress. These CGs were separated in classes: (i) Genes related to ABA synthase and signaling pathway such as Ca*NCED, CaPYL8a, CaPYL8b, CaPYR1, CaSNRK2.8* and *CaSNRK2.10* and *CaAHG3* encoding a 9-cis-epoxycarotenoid dioxygenase, ABA receptors and proteins phosphatase, respectively. (ii) genes encoding for transcription factors ABA-independent and -dependent such as *CaERF017*, *CaEDR2* e *CaMYB (*iii*)* genes coding for different ascorbate peroxidase isoforms (*CaAPX1*, *CaAPX5* e *CaAPX6)* which ROS removing enzyme catalyzes the conversion of H_2_O_2_ to H_2_O*; (iv)* genes encoding for a galactinol synthase *CaGolS,* that catalyzes the key step in Raffinose Family of Oligosaccharides biosynthesis; dehydrin (*CcDH1*), and *CaSDD1* encoding a subtilisin-like serine protease involved stomatal density and distribution.

Expression analyses and characterization of these candidate genes associated with drought tolerance may lead to the identification of marker polymorphisms with a great potential to assist in the identification and early selection of drought-tolerance coffee genotypes. This, certainly, can have an impact on coffee breeding programs, due to time savings and optimization of resources for the development of drought tolerant cultivars. Currently, the rainfed cultivation system is dominant in small-farmers coffee production and, therefore, the selection and characterization of drought tolerant progenies may lead to new cultivars which could guarantee lower risks and higher income for these coffee farmers. The development of drought-tolerant coffee cultivars may also lead to a better management of natural resources (water) allowing coffee to continue to be produced without irrigation or even reduce its consumption under irrigated systems. It should be emphasized that the progenies studied on this work, also have a rust resistance phenotype, which reduces the production costs and the risks to the environment and rural workers, as less pesticides would be required.

The aim of this study was to identify the correlation between photochemical efficiency and candidate genes expression to elucidate the drought tolerance mechanisms in coffee progenies (Icatu Vermelho IAC 3851-2 × Catimor UFV 1602-215) previously identified as tolerant in field conditions.

## Results

For this study, three progenies potentially drought tolerant (progenies 5, 12 and 2) and one sensitive (progeny 15) were previously selected in the field, where the average productivity of the most productive progenies (progenies 5, 12 and 2) was 70.00 bag ha^−1^ and the vigor score was 10. The progeny 15, on the other hand, showed productivity of 34.00 bag ha^−1^ and the vigor score was 5.

Leaf Ψpd with values below − 1.0 MPa confirmed the unstressed condition of irrigated plants (Table 1). In this condition, no differences were observed between the progenies in relation to the values of net CO_2_ assimilation rates (A), stomatal conductance (gs), transpiration (E), vapor pressure deficit (VPD) and leaf temperature (Tleaf), quantum yield of PSII electron transport (ΦPSII), ETR photochemical quenching, non-photochemical quenching (qN) and the NPQ. For variables A, gs and E there was an increase in their values in the second evaluation followed by a slight decrease in the last, while for the variables VPD and Tleaf, highest values were observed at 35 days after irrigation withdraw. The progeny 15 presented lower leaf water potential compared to the other progenies, reaching values below − 5.0 MPa at 28 and 35 days after irrigation withdraw. It is worth to mention that at 35 days, progeny 12 reached the water potential of − 3.35 MPa, while progeny 2 and 5 maintained higher values of water potential, being 1.30 and 1.63 MPa, respectively (Table 1). The values of net CO_2_ assimilation rates (A), stomatal conductance (gs), and transpiration (E) were lower under water deficit conditions than irrigated controls for all progenies at 28 and 35 days. For progeny 15, the values of A, gs and E were 82%, 93% and 90% lower than the irrigated control at 35 days. When comparing the progenies, there were statistical differences using the Duncan test, highlighting progeny 15 with lower values, especially in relation to progeny 2.

**Table 1:** Pre-dawn water potential (Ψpd), photosynthetic rate (A), stomatal conductance (gs), transpiration (E), vapor pressure deficit (VPD) and leaf temperature (Tleaf) of 4 irrigated (I) and non-irrigated (NI) progenies at 0, 28 and 25 days of evaluation.

Progeny	0 days		28 days		35 days	
	I	NI	I	NI	I	NI
**Ψpd (MPa)**						
2	− 0.73 ± 0.07 aA	− 0.30 ± 0.03 aA	− 0.47 ± 0.05 aA	− 0.78 ± 0.12 aA	− 0.35 ± 0.03 aA	− 1.30 ± 0.25 aB*
5	− 0.32 ± 0.04 aA	− 0.50 ± 0.02 aA	− 0.37 ± 0.04 aA	− 1.17 ± 0.04 a B*	− 0.38 ± 0.04 aA	− 1.63 ± 0.22 aB*
12	− 0.62 ± 0.03 aA	− 0.53 ± 0.04 aA	− 0.70 ± 0.12 aA	− 1.02 ± 0.13 aB*	− 0.36 ± 0.05 aA	− 3.35 ± 0.58 bC*
15	− 0.73 ± 0.03 aA	− 0.50 ± 0.04 aA	− 0.40 ± 0.04 aA	− 5.97 ± 0.05 bB*	− 0.36 ± 0.04 aA	− 5.88 ± 0.10 cB*
**A(µmol CO**^**2**^** m**^**−2**^** s**^**−1**^**)**						
2	6.315 ± 1.701 aB	3.945 ± 0.519 aA	11.188 ± 1.412 aA	5.457 ± 0.927 aA*	8.490 ± 0.360 aAB	3.793 ± 0.326 aA*
5	7.084 ± 1.683 aA	4.633 ± 0.951 aA	10.210 ± 2.183 aA	3.759 ± 0.154 abA*	8.095 ± 1.835 aA	2.463 ± 0.171 abA*
12	5.048 ± 0.215 aB	4.243 ± 1.138 aA	9.642 ± 0.992 aA	4.583 ± 0.886 abA*	8.314 ± 1.727 aAB	2.113 ± 0.346 abA*
15	4.299 ± 0.546 aB	5.249 ± 1.273 aA	10.626 ± 1.416 aA	2.886 ± 0.193 bA*	8.049 ± 0.638 aAB	1.401 ± 0.358 bA*
**gs (mol H**_**2**_**O m**^**−2**^** s**^**−1**^**)**						
2	0.097 ± 0.046 aB	0.040 ± 0.002 aA	0.179 ± 0.044 aA	0.043 ± 0.005 aA*	0.071 ± 0.003 aB	0.020 ± 0.003 aA*
5	0.080 ± 0.011 aA	0.042 ± 0.012 aA	0.140 ± 0.037 aA	0.033 ± 0.005 abA*	0.079 ± 0.029 aA	0.013 ± 0.003 abA*
12	0.067 ± 0.013 aA	0.050 ± 0.015 aA	0.135 ± 0.030 aA	0.042 ± 0.11 abA*	0.092 ± 0.025 aB	0.008 ± 0.001 abA*
15	0.047 ± 0.012 aB	0.069 ± 0.019 aA	0.164 ± 0.043 aA	0.028 ± 0.001 bA*	0.072 ± 0.011 aB	0.005 ± 0.001 bB*
**E (µmol H**_**2**_**0 m**^**−2**^** s**^**−1**^**)**						
2	1.093 ± 0.322 aB	0.684 ± 0.099 aA	2.753 ± 0.448 aA	0.886 ± 0.124 aA*	1.663 ± 0.096 aB	0.627 ± 0.126 aA*
5	1.224 ± 0.232 aB	0.638 ± 0.117 aA	2.376 ± 0.622 aA	0.678 ± 0.125 a A*	1.597 ± 0.558 aAB	0.358 ± 0.086 abA*
12	0.981 ± 0.238 a B	0.755 ± 0.166 aA	2.234 ± 0.387 aA	0.857 ± 0.201 aA*	1.807 ± 0.400 aAB	0.186 ± 0.015 abA*
15	0.639 ± 0.079 aC	1.025 ± 0.276 aA	2.449 ± 0.518 aA	0.615 ± 0.062 aA*	1.585 ± 0.160 aB	0.150 ± 0.014 bA*
**VPD (kPa)**						
2	1.348 ± 0.201aB	1.489 ± 0.149aA	1.469 ± 0.113aB	1.806 ± 0.089aAB	2.103 ± 0.085aA	2.236 ± 0.376aA
5	1.316 ± 0.103aA	1.597 ± 0.209aB	1.522 ± 0.031aA	1.798 ± 0.080aB	1.815 ± 0.118aA	2.394 ± 0.308aA
12	1.339 ± 0.188aB	1.457 ± 0.148aB	1.534 ± 0.102aB	1.846 ± 0.134aAB	2.004 ± 0.200aA	2.317 ± 0.376aA
15	1.390 ± 0.191aB	1.474 ± 0.187aB	1.463 ± 0.126aB	1.906 ± 0.130aAB	2.041 ± 0.155aA	2.263 ± 0.367aA
**T leaf (°C)**						
2	25.2 ± 0.959aC	25.8 ± 1.150aC	29.4 ± 0.347aB	29.9 ± 0.592aB	31.9 ± 0.311aA	31.8 ± 1.072aA
5	25.0 ± 0.753aC	26.1 ± 1.148aC	29.3 ± 0.264aB	29.9 ± 0.569aB	31.2 ± 0.419aA	32.4 ± 0.795aA
12	24.9 ± 1.079aC	25.7 ± 1.120aB	29.4 ± 0.362aB	30.0 ± 0.668aB	31.9 ± 0.600aA	32.2 ± 0.894aA
15	25.1 ± 0.990aC	25.7 ± 1.137aC	29.3 ± 0.347aB	30.3 ± 0.754aB	31.9 ± 0.510aA	32.0 ± 1.041aA

Means followed by the same lowercase letter in the column and averages followed by the same capital letter in the row in each irrigation condition do not differ statistically from each other at the 5% probability level by the Duncan test. Means followed by asterisks in the line, for the same time and even progeny, are statistically different at the 5% probability level by the Duncan test.

**Figure 1:**
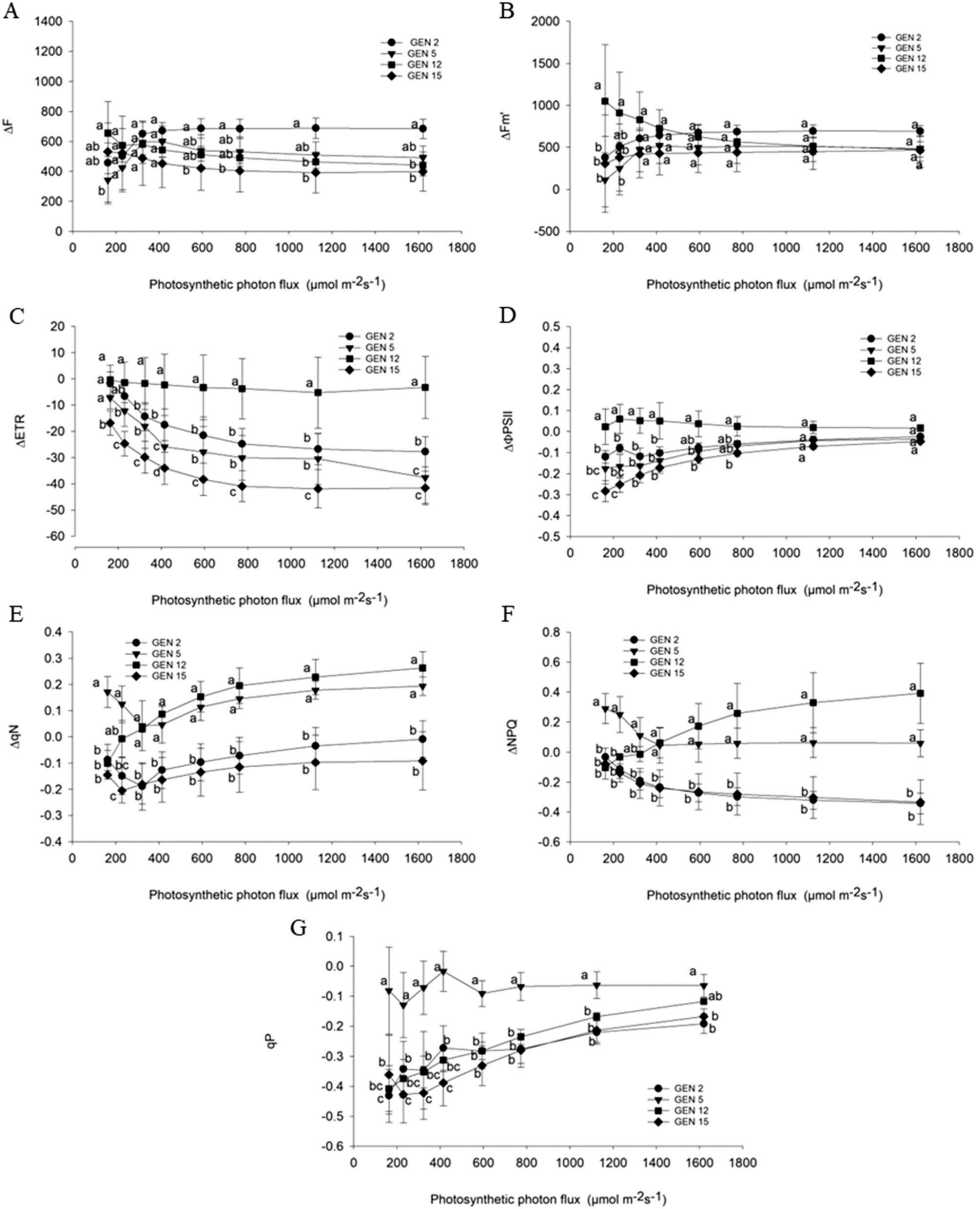
Response curve of the minimum estimated fluorescence of leaves adapted to light (F) (**A**), of the maximal fluorescence (Fmʹ) (**B**), the relative rate of electron transport (ETR) (**C**), effective photochemical efficiency of PSII (ΦFSII) (**D**), non-photochemical quenching (qN) (**E**), the non-photochemical extinction coefficient (NPQ) (**F**), photochemical quenching (qP) (**G**) to the increase of the photosynthetic photon flux density (DFFF) in arabica coffee progenies at 35 days of evaluation.

In relation to the parameters ΔF and ΔFmʹ, positive values were found indicating an increase of F and Fmʹ under water deficit (Fig. 1A,B). From the flux density of photosynthesizing photons of 600 μmol m^−2^ s^−2^, progeny 2 presented higher ΔF while the lowest value was found in progeny 15. As for ΔFmʹ, the progeny 12 presented the highest value when submitted to 162 to 230 μmol m^−2^ s^−2^, as compared to the other progenies.

Considering the ΔETR, the curves presented negative values, which shows that under water deficit, the ETR of the progenies were lower than when irrigated (Fig. 1C). We should draw attention to progeny 15, which presented a more negative ΔETR, and the progeny 12 with ΔETR values close to zero. Of these, progeny 15 presented a more expressive reduction of ETR and progeny 12, a small magnitude of reduction. For progenies 5 and 2, the reduction was intermediate.

As for ΔɸPSII, corresponding to the variation of the effective quantum yield, progeny 12 presented a variation close to zero, indicating that the deficit did not reduce the ɸPSII values, as compared to the irrigated one (Fig. 1D). In the other progenies, the values of the curves were negative and, therefore, the effective quantum yield under water deficit was lower in these progenies. With the increase in the density of photosynthetic photon flux, the differences between the progenies were less pronounced, with no differences occurring at 1100 μmol m^−2^ s^−2^.

In general, with increasing photosynthesizing photon flow density, progenies 5 and 12 presented positive values of ΔqN and ΔNPQ, indicating higher qN and NPQ, when submitted to water deficit (Fig. 1e,f). In contrast, progenies 15 and 2 presented negative values of ΔqN and ΔNPQ, therefore with reduction of these parameters under water deficit. The values of ΔqP were negative indicating a reduction of qP under deficit, but the progeny 5, which presented higher values of delta, was observed, indicating that the qP was less affected in this progeny as compared to the others (Fig. 1g).

**Figure 2:**
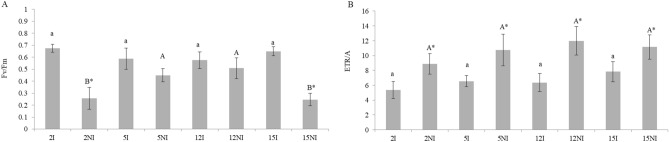
Maximum photochemical efficiency of PSII (Fv/Fm ratio) (**A**) and electron transport-to-net photosynthesis rate ratio (ETR/A) (**B**) of 4 irrigated (I) and non-irrigated (NI) progenies at 35 days of evaluation. Ranked results were analyzed using the non-parametric Kruskal–Wallis test followed by a pairwise comparison with the Mann–Whitney test. Significance level were established as p-values < 0.05, for both tests. Means followed by the same lowercase letter and averages followed by the same capital letter in each irrigation condition do not differ statistically from each other. Means followed by asterisks for even progeny are statistically different.

Regarding the maximum photochemical efficiency of PSII (evaluated by the Fv/Fm ratio), similar values were observed under irrigated and non-irrigated conditions for progenies 5 and 12 (Fig. 2A). However, the Fv/Fm ratio was lower in the non-irrigated condition compared to the irrigated condition in progenies 15 and 2, with lower values in relation to progenies 5 and 12. In the drought-stressed progenies, the ETR/A ratio enhanced remarkably. However, there were no significant differences between them (Fig. 2B).

**Figure 3:**
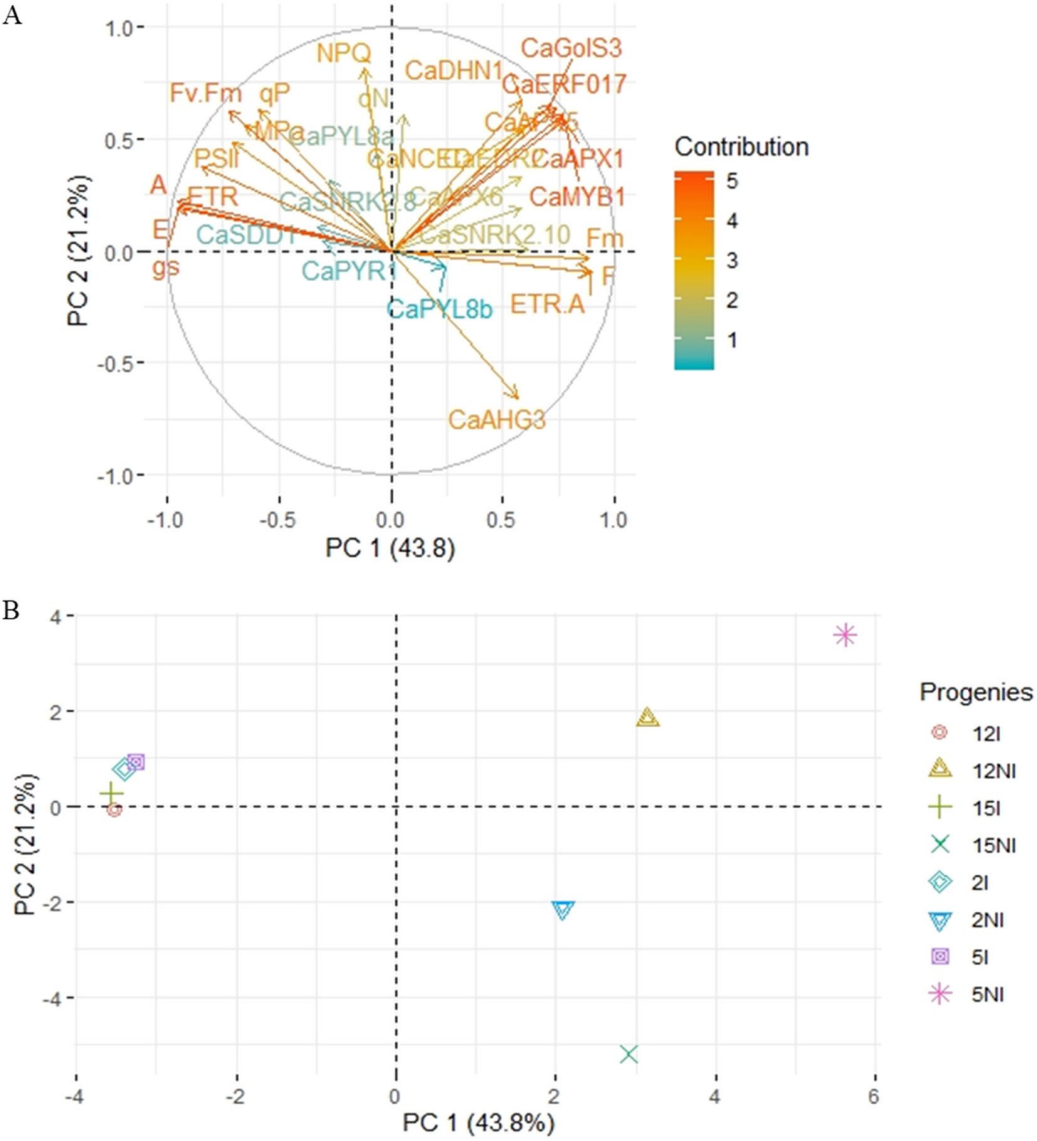
The first two axes of a PCA of all 4 irrigated (I) and non-irrigated (NI) progenies. The size of the vectors (arrows) represents the correlation between variable and PC. The plots show the contribution of the physiological characteristics and several gene expression profile (**A**) and segregation scores of each progeny under irrigated and non-irrigated conditions (**B**) for the first two principal components.

In order to verify the correlation between physiological characteristics and gene expression, a statistical analysis was performed, using PCA, at 35 days, when plants effectively reached a water stress (Fig. 3). The analyses considered the two PCs corresponding to the two eigenvalues with the highest values, which explained more than 65% of the variance. Figure 3A shows a greater variance explained by the PC1 for positively associated variances with *CaMYB1, CaAPX1, CaGolS3, CaERF017, CaDHN1, CaNCED, CaAPX5* and *CaEDR2* and negatively with the physiological characteristics. The PC1 was able to differentiate irrigation regimes, with higher values of gene expression for non-irrigated treatments. On the other hand, in PC2 the variables of greatest contribution were *CaMYB1, CaAPX1, CaGolS3, CaERF017, CaDHN1, CaNCED, CaAPX5, CaEDR2* and *CaPYL8a,* together with the fluorescence variables. In this case, progenies 12 and 5 under water deficit conditions were discriminated together with the progenies under irrigated conditions (Fig. 3B). According to the PCA, gene expression analyses will be focused and detailed for the genes that contributed to the formation of PC2, since they were decisive in distinguishing progenies 5 and 12 that showed the greatest photochemical efficiency.

**Figure 4:**
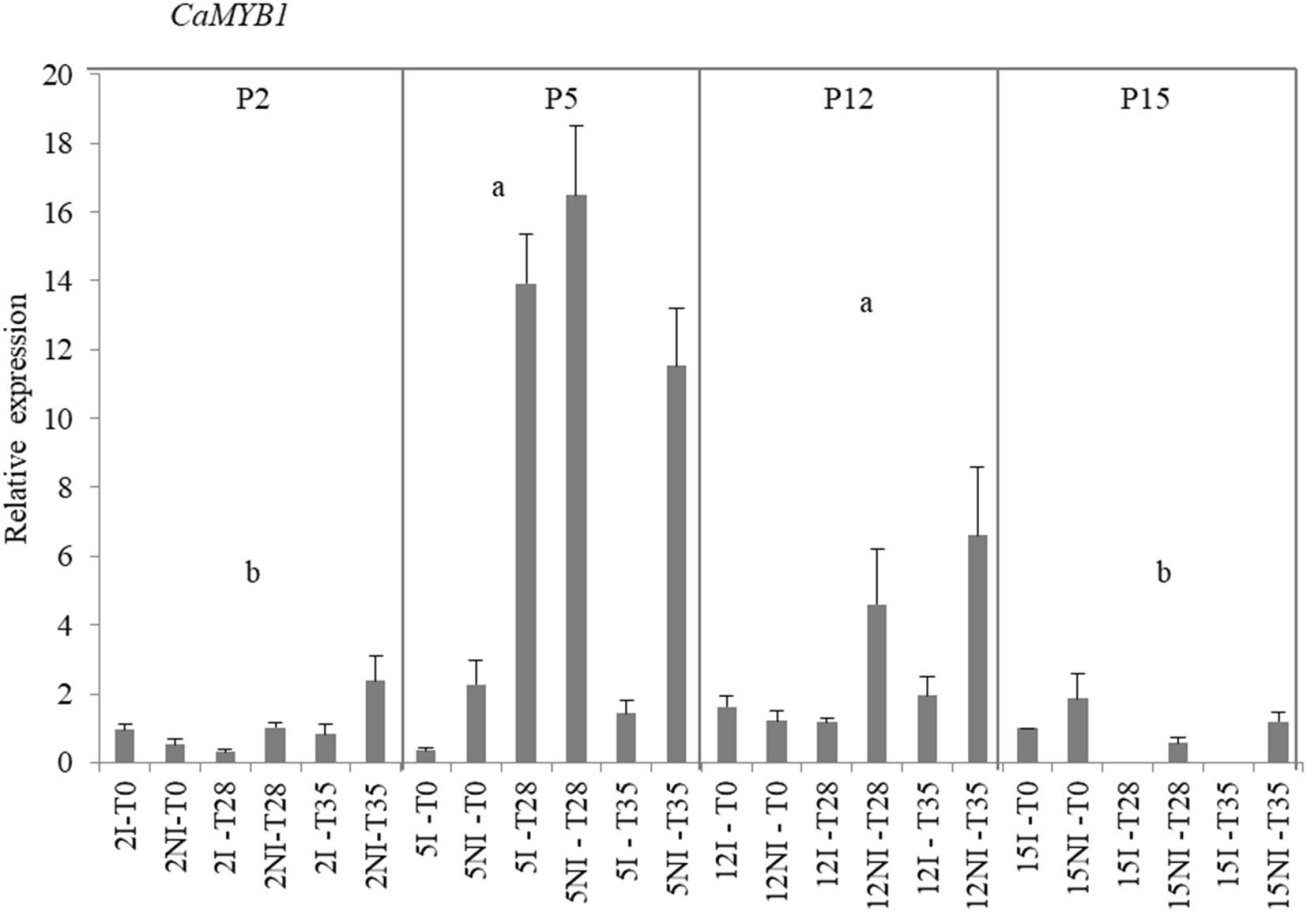
Gene expression profile under irrigated (I) and non-irrigated (NI) conditions. Gene expression of the *CaMYB1* gene was analyzed on leaves of coffee progenies 2, 5, 12 and 15. Abundance of transcripts was normalized using *CaUBQ10* gene expression as an endogenous control. The results were expressed using 15 I as the reference sample (Relative Expression = 1). Values of three replicates were presented as means ± SE (bars). Ranked results of the relative expression data of the progenies were analyzed using the non-parametric Kruskal–Wallis test followed by a pairwise comparison with the Mann–Whitney test. Significance level were established as p-values < 0.05, for both tests. Progeny patterns of relative expression values followed by the same lowercase letter do not differ statistically from each other.

A first univariate analysis for the expression data of candidate genes, all the treatments (time and condition) were grouped by progeny and analyzed statistically. The only significant difference among the expression patterns of the different progenies was found for the *CaMYB1* (Fig. 4) In this case, the pattern of relative expression, among all treatments, displayed by the progenies 5 and 12, was statistically different from progenies 2 and 15 (Fig. 4).

**Figure 5:**
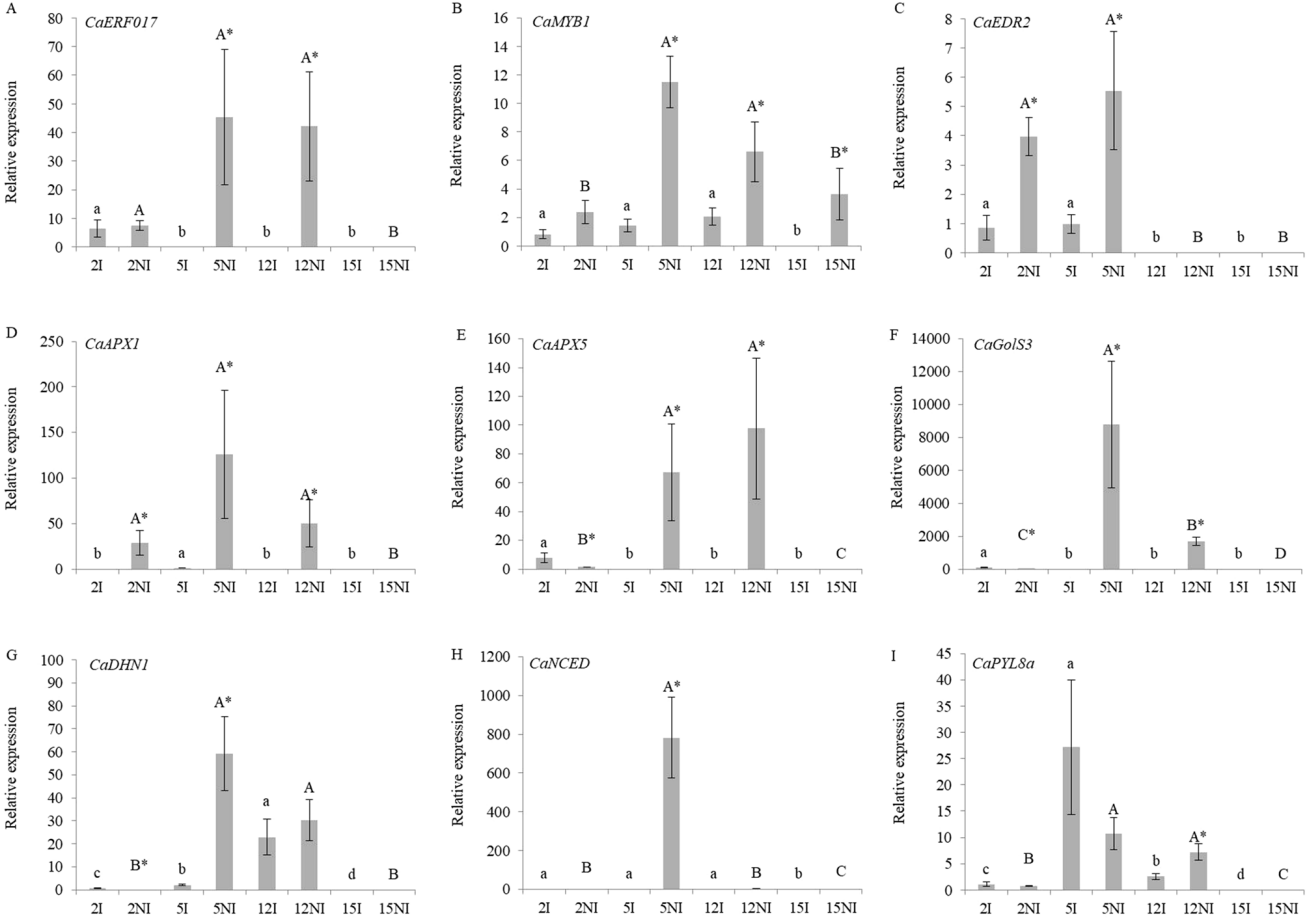
Gene expression profile under irrigated (I) and non-irrigated (NI) conditions. Gene expression was analyzed on leaves of 2, 5, 12 and 15 progenies. (**A**) *CaERF017* gene expression; (**B**) *CaMYB1* gene expression; (**C**) *CaEDR*2 gene expression; (**D**) *CaAPX1* gene expression; (**E**) *CaAPX5* gene expression; (**F**) *CaGolS3* gene expression; (**G**) *CaDHN1* gene expression; (**H**) *CaNCED* gene expression; (**I**) *CaPYL8a* gene expression. Abundance of transcripts was normalized using *CaUBQ10* gene expression as an endogenous control. The results were expressed using 15 I as the reference sample (Relative Expression = 1). Values of three replicates were presented as means ± SE (bars). Ranked results of the relative expression data of the progenies were analyzed using the non-parametric Kruskal–Wallis test followed by a pairwise comparison with the Mann–Whitney test. Significance level were established as p-values < 0.05, for both tests. Means followed by the same lowercase letter and averages followed by the same capital letter in each irrigation condition do not differ statistically from each other. Means followed by asterisks for even progeny are statistically different.

The second analysis was performed only with the gene-expression data of the 35 days condition as it was done for the PCA analysis. The obtained results with the 35 day’s data analysis among the treatments and progenies showed that statistical differences were observed for the expression data of all genes studied. The results of the analyses of the expression profiles of some transcription factors such as *CaERF017*, *CaMYB1* and *CaEDR*2 are presented on Figs. 5A–C. For *CaERF017* an expressive expression was found at 35 days, with higher values found under non-irrigated progenies 2, 5 and 12 and lower values under both, irrigated and non-irrigated progeny 15 (Fig. 5A). For the *CaMYB1* gene- expression levels, progenies 5 and 12 presented the highest expression levels under the non-irrigated condition (Fig. 5B). However, as shown on Fig. 3, high levels of *CaMYB1* expression were also observed for progenies 5 and 12, at the time point of 28 days. For the *CaEDR2* gene, at 35 days, progenies 2 and 5 presented higher expression levels under both conditions, as compared to the other progenies, with significant higher values under the non-irrigated condition as compared to the irrigated (Fig. 5C).

For *CaAPX1*, expression was up-regulated in progenies 2, 5 and 12 under the non-irrigated condition (Fig. 5D). Furthermore, for *CaAPX5* transcript levels, higher expression values were found for non-irrigated progenies 5 and 12 (Fig. 5E). Higher values of relative expression of *CaGolS3* were observed in progeny 5 followed by 12, both under the non-irrigated condition, as compared to the other treatments studied (Fig. 5F).

Results of the *CaDHN1,* shows that the highest value of relative expression was observed for progeny 5 in the non-irrigated condition, whereas for progeny 12 there was no differential expression between irrigated and non-irrigated condition (Fig. 5G). The highest value of relative expression for the *CaNCED* gene was presented by progeny 5 non-irrigated at 35 days (Fig. 4H). The *CaPYL*8a gene displayed the higher values for progenies 5 and 12 for the non-irrigated condition as compared to the other treatments (Fig. 5I).

Noteworthy that for all genes tested, as shown on Fig. 5A–I, progeny 15 displayed the lowest levels of gene expression.

## Discussion

In Brazil, the damaging effects of water deficit are enhanced by the high irradiances usually observed in coffee-growing regions^4^. The coffee tree has a decreased stomatal conductance (gs) under water stress, which leads to a huge oxidative pressure^26,27^. Therefore, progenies which can undertake adjustments in photochemical and molecular processes might better endure drought stress conditions. In this study, we sought to understand about the strategies these different progenies use in order to avoid photochemical damaging.

The results presented here indicated that coffee progenies 2, 5 and 12 that presented high productivity under field conditions in a year of severe water deficit, considered potentially tolerant, showed a different pattern of photochemical and molecular responses to the drought conditions. Progeny 5 showed capacity to maintain water potential, which contributed to lower qP variation and gas exchange between irrigated and deficit conditions, proving the drought tolerance condition observed in the field. However, qN and NPQ increases in response to the water stress indicate that this progeny is photochemically responsive to small variations of Ψpd protecting the photosystem and maintaining qP. As qP represents the portion of absorbed energy that is used in the photochemical step by photosystem II reaction centers^17^, it may also be indicative of the proportion of reaction centers that are open^13^. The highest values of the non-photochemical quenchings qN and NPQ protect the reaction centers of photosystem II by dissipating excess energy in the form of heat^28,29^. This was also evident from values of Fv/Fm that remained unchanged in stressed plants, indicating that there was no photoinhibition in this progeny and that light reactions were in a fully active state^26^.

Among the tolerants, progeny 12 presented lower water status maintenance capacity, as verified in the lower values of Ψpd. It should be noted, however, that the reduction of gas exchange under water deficit showed similar levels to those progenies that maintained the leaf-water potential. In spite of having reduced qP under water deficit, the increase of qN and NPQ provided maintenance of the photochemical parameters as ɸPSII and ETR. This shows that this progeny has a certain tolerance of the photosynthetic apparatus to a severe water deficit (Ψpd =  − 3.35 MPa). The stress condition may lead to a slight increase in excitation pressure at the reaction center in the PSII^30^, as indicated by the decrease in qP, however, not being sufficient to decrease the ETR, which in turn reflects activities of photosystem II^28^ and the effective photochemical efficiency of PSII (ɸPSII). The maintenance of Fv/Fm values in this progeny under drought also highlights that there were no PSII inhibitions^26^. Similar results were found by Li et al.^28^ that observed under drought an increase of qN and decrease of qP in sugar beet leaves, suggesting that excess energy was dissipated as heat, which prevented a photochemical inhibition, due to a decreased degree of reaction-centers opening.

On the other hand, progeny 2, even with higher Ψam, proved to be photochemically sensitive, since there was a greater reduction in qP, ETR, qN, NPQ and Fv/Fm under water deficit. These results indicate that the photosynthetic apparatus may have been damaged by photoinhibition and that it was unable to protect itself against excess energy^26,28,31^.

The progeny 15 exhibited sensitivity to drought, as previously noted under field condition as this progeny had the lowest productivity in the year of occurrence of a severe drought. It presented the lowest capacity of water potential (Ψpd) maintenance after 28 days of water stress, due to the higher decay rate of this variable among the progenies studied. Progeny 15 showed photochemical sensitivity, with reduction of all fluorescence parameters under water deficit. There is a decrease in CO_2_ availability for ribulose-1,5-bisphosphate carboxylase/oxygenase (RuBisCO) due to stomatal closure induced by drought. This may causes an excess of energy in the chloroplasts that can lead to an increase in ROS levels, increasing oxidative stress^7^. Thus, drought stress effects on the photosynthetic apparatus of this progeny has typically started with stomatal effects, and ended in metabolic changes caused by severe stress^32^. It is emphasized that the plants were not exposed to different levels of oxidative pressure since there were no differences among the progenies in ETR/A ratio within the same system. However, a high ETR/A ratio was observed in all progenies under drought stress, which are likely associated with an increase in reducing power that might lead to oxidative stress^27^. In progeny 15 drought-stressed, a decrease in Fv/Fm was found in relation to well-watered plants, suggesting that effective oxidative damage occurred in these plants. It was also observed that in South African coffee cultivars lower values of Fv/Fm were found in drought-sensitive cultivars^26^. Also, the agronomic vigor analysis showed that progenies that didn’t present effective photochemical adjustments had lowest agronomic vigor, marked depletion symptoms and yellowing (leaf scald).

Overall, progenies 5 and 12 showed high photochemical efficiency and energy dissipation capacity, analyzed by the parameters of the chlorophyll a fluorescence curves. Tounekti et al.^26^ studying South African coffee cultivars confirmed that photoprotection is an important factor that affects photosynthetic productivity, and that it varies between the cultivars. Interestingly, the PCA analysis revealed that the expression levels of *CaMYB1, CaAPX1, CaGolS3, CaERF017, CaDHN1, CaNCED, CaAPX5*, *CaEDR2* and *CaPYL8a* genes clearly displayed the major response to drought stress and discriminated these progenies (5 and 12) under the water-deficit conditions. Accordingly, the gene expression analysis showed that for *CaMYB1, CaERF017*and *CaEDR2*, transcript levels coding for these regulatory proteins were higher in the tolerant progenies 5 and 12 as compared to the others. In *C. arabica CaERF017* gene was identified as *C canephora* DREB-like gene^25^. The DREB (Dehydration Responsive Element Binding) gene family corresponds to key transcription factors involved in responses to various abiotic stresses, which regulate the expression of various responsive genes, acting downstream of ABA-dependent and -independent signal transduction pathways^33,34^. The *CaERF017* gene was the most expressed DREB-like gene in leaves under low humidity in the drought tolerant clones^25^. The MYB transcription factors have been reported by regulating the biosynthesis of secondary metabolites, regulation of lateral root growth and of the stomatal movements^35,36^. The *EDR2* gene encodes a protein that acts as a negative regulator of cell death caused by the attack of pathogens and mediated by salicylic acid^37^.

Among ABA-related genes, *CaNCED* expression highlighted the progeny 5 without irrigation. NCED catalyzes the cleavage of 9-cis-xanthophylls to xanthoxin, which is the key regulation setp of ABA biosynthesis in plants^38^. Accumulation of this hormone can trigger the expression of many stress-responsive genes increase the tolerance of the plant^39^. In *C. canephora* the tolerant clone 14 showed expression 4 times larger than the other clones in response to drought. *NCED* gene expressions were strongly induced in response to water stress in tolerant progenies of several common bean progenies^40,41^. The ABA synthesized is perceived by ABA receptors as the PYRABACTIN RESISTANCE1 (PYR1)/PYR1-LIKE (PYL)/REGULATORY COMPONENTS OF ABA RECEPTORS (RCAR) family of proteins^42,43^. Greater expression of *CcPYL3* and *CcPYL7* was detected in tolerant clones of C. canephora, but with a differential expression between them^21^ as observed in this study for *C. arabica* with greater expression in progenies 5 and 12 under drought.

The high levels of gene expression encoding isoforms of the antioxidant enzyme ascorbate peroxidase in progenies 5 and 12, mainly under water deficit condition, may be related to the maintenance of the gas exchanges and greater photochemical efficiency in these progenies under non-irrigated condition. The excess excitation energy can lead to the production of reactive oxygen species (ROS) that can oxidize lipids, proteins and nucleic acids in water deficit condition^44^. The peroxidase enzyme ascorbate (APX) plays a key role in the removal of ROS, catalyzing the conversion of H_2_O_2_ to H_2_O, using ascorbate with the electron donor^45^. Thus, increased expression of enzymes antioxidants may provide greater protection of photosystem II and other membranes to oxidative stress, contributing to efficiency of the photochemical phase of photosynthesis in these genotypes that showed the highest values of Fv/Fm.

Similarly, the *CaGolS3* expression was detected with higher levels in the progenies 5 and 12, corroborating with the literature that shows that in *C. arabica* the GolS3 isoform is highly expressed under moderate and severe drought conditions^46^. In *C. canephora* a fourfold higher expression was also observed in the tolerant clone 14 under drought condition^18^. *CaGolS3* gene encodes an isoform of the enzyme galactinol synthase that is involved in the first step of oligosaccharide synthesis in the raffinose (RFOs) Family. RFOs are involved both in osmoprotection as in ROS scavenging, thus promoting increased protection the photosynthetic apparatus against oxidative damages caused by various stresses such as drought^47^. On the other hand, the lower levels of expression of these genes in progeny 2 may have contributed to the decrease of all fluorescence values, perhaps because of a less effective protective and antioxidant action.

Noteworthy is also that the *CaDHN1* gene expression was found in non-irrigated plants mainly in progenies 5 and 12. Dehydrins are known as a group II of LEA proteins (Late embryogenesis abundant), accumulated in the later stages of development in response to the most diverse environmental stresses, participating significantly in the stabilization of membranes, enzymes and nucleotides^48^. The expression of dehydrins can be strongly induced by abiotic stresses^49,50^, being more expressed in tolerant clones^18,22^.

In our study, the better photochemical efficiency and greater capacity to dissipate unused energy in photosynthesis, was clearly associated with higher gene expression levels coding for regulatory proteins, ABA-synthesis, cellular protectors and isoforms of the antioxidant enzyme ascorbate peroxidase, indicating that these genes can contribute to maintaining the integrity of the photosynthetic apparatus in progenies tolerant to water deficit. On the other hand, progeny 15 showed photochemical sensitivity, as well as the lowest expression levels of almost all genes tested. In addition these results are also in agreement with the lowest productivity of progeny 15, observed under field conditions in a year of a severe drought period.

Progenies with better photochemical efficiency and greater capacity to dissipate unused energy in photosynthesis may present better condition to maintain its function under low leaf water status and to recovery after a period of severe drought, under a large scale of radiation intensity.

## Material and methods

### Plant material

The *Coffea arabica* progenies were selected by the coffee breeding program conducted and coordinated by Agricultural Research Enterprise of Minas Gerais (EPAMIG, Lavras, Minas Gerais, Brazil) with the participation of the Universidade Federal de Lavras (UFLA) and the Universidade Federal de Viçosa (UFV). The four progenies in the F6 generation (2 (H 29-1-8-5-14-2), 5 (H 136-1-19-7-14-4), 12 (H 136-1-19-4-6-5) and15 (H 30-3-14-1-19-9-12)) were selected in an experiment at the EPAMIG Experimental Field of São Sebastião do Paraiso (CESP, Minas Gerais, Brazil) from ‘Icatu (3851-2-UFV 2117) and Catimor UFV 5373’ crossing, based on data on productivity and agronomic vigor observed in 2014/2015 crop year (a period with severe water deficits in this region). According to the EPAMIG genetic improvement program database, three more productive genotypes with maximum agronomic vigor (progenies 5, 12 and 2) and one less productive and with lowest agronomic vigor (progeny 15) were selected. Plants were scored according to a ten point arbitrary scale in which score 1 corresponds to plants with the poorest vegetative vigor and marked depletion symptoms. The score 10 indicates plants with excellent vigor, more leafy and with marked vegetative growth of the productive branches^51,52^. Productivity is measured by weighing the fruit immediately after harvest. Then a sample of about 3 L from each plot is weighed and placed for drying in the sun. After drying, the coffee is weighed, processed and weighed again to calculate productivity in processed bags per hectare (bag ha^−1^).

The use of coffee progenies in the present study has the appropriate permissions and/or licenses of the EPAMIG coffee breeding program. The use of coffee plants parts complies with international, Brazilian and institutional guidelines.

### Experimental procedures

The experiment was carried out in a greenhouse with free exchange of air located in the latitude of 21° 14′ 30′′ South and longitude of 45° 00′ 10′′ West and altitude of 918,841 m (Main Climatological Station of Lavras). The minimum, average and maximum temperatures and relative humidity and photosynthetically active radiation of the greenhouse during the experimental period were 17 °C, 26 °C, 35 °C, 39%, 63%, 87% and 300 µmol m^−2^ s^−1^, respectively.

The seedlings were grown in 20 L polyethylene pots, with a substrate consisting of a mixture of subsoil soil, sand and bovine manure (3: 1: 1, v/v/v). The fertilization was carried out according to the substrate analysis, following the recommendations of Guimarães et al.^53^. Phytosanitary treatments were carried out preventively to control the main pests and diseases of higher incidence in the region. After being transferred to the pots, the seedlings were kept in a greenhouse, with free exchange of air during a period of eight months.

Until the day before the application of the water treatments all plots were irrigated in order to maintain the substrate of the vase in the field capacity (FC). In order to control the amount of water applied in each vessel, the humidity sensor (ML2X THETA PROBE, Delta-T Devices) was used and calibrated according to soil water moisture, from soil saturation to field capacity.The relative soil moisture at 100% field capacity was 0.46 m^−3^ m^−3^. After the eight-month period, the plants were separated into two groups. In the first group (i.e., irrigated plants (I), control), plants remained irrigated, maintaining 100% of the field capacity. The plants from the second group (i.e., drought-stressed plants (NI)) were not watered until the soil water content reached approximately 35% FC (i.e., 0.16 m^−3^ m^−3^; ca. 28 days). They were then maintained at 35% FC for additional 7 days, (i.e., 0.16 m^−3^ m^−3^; ca. 35 days) in all progenies.

### Experimental layout

The experiment was set up in a randomized complete block design (RCBD) with split split plot in time. The experiment consisted of 8 treatments, in a 4 × 2 factorial scheme, four progenies and two water treatments. The subplots were the different epochs of evaluation (0, 28 and 35 days after the beginning of the experiment). Four replicates were used and each experimental plot consisted of one plant. In total, we used 32 plants, 8 per progeny (4 irrigated, 4 non-irrigated).

### Statistical analyses

Physiological traits were evaluated by analysis of variance using the F test, with the Duncan’s New Multiple Range Test (DNMRT) at 5% probability for to perform the mean differences using the R software^54^ and agricolae package^55^. The physiological traits and gene expression profiles were also analyzed by Principal Component Analysis (PCA), using the R software^54^ and the FactoMineR package^56^.

For the RT-qPCR results, Fv/Fm and ETR/A, ranked results were analyzed using the non-parametric kruskal–Wallis test followed by a pairwise comparison with the Mann–Whitney test. Significance levels were established as p-values < 0.05, for both tests. The spreadsheet software Excel was used to perform the analyses.

### Physiological traits

All samplings and measurements were made using leaves from the third or fourth pair of apex plagiotropic branches.

Leaf samples for pre-dawn water potential (Ψpd) measurements were collected between 3 and 5 am. The collected leaves were placed in plastic bags, properly closed and identified according to each treatment. Then the samples were placed in a thermal box with ice, to avoid excessive water loss, and taken to the place where Ψpd measurements were made using the Scholander-type pressure chamber (PMS Instruments Plant Moisture-Model 1000).

The net photosynthetic rate (A—μmol CO^2^ m^−2^ s^−1^), stomatal conductance (gs—molH_2_O m^−2^ s^−1^), transpiration (EmmolH_2_O m^−2^ s^−1^), vapor pressure déficit (VPD—kPa) and leaf temperature (Tleaf—°C) were measured between 8:00 and 11:00 am under an artificial source of photosynthetically active radiation (PAR), in a closed chamber fixed in 1500 μmol of photons m^−2^ s^−1^ (Blue + Red LED LI-6400-02B, LI-COR, Lincoln, USA) with a infrared gas analyzer (LI-6400XT Portable Photosynthesis System, LICOR, Lincoln, USA). The CO_2_ assimilation rate in the chamber was measured with the ambient CO_2_ concentration.

Chlorophyll fluorescence was measured with a pulse amplitude modulation fluorometer (MINI PAM-1999, Heinz Walz GmbH, Effeltrich, Germany). The analyzes were carried out following the methodology described by Schreiber et al.^57^ and Genty et al.^58^ and the terminologies and calculations proposed by Snel and Van Kooten^59^. Leafclips were used for measurements of the minimum (F0) and maximum (Fm) fluorescence yield in dark-adapted (30 min) leaf tissues. The minimum fluorescence (F0) was measured using a weak modulated measuring beam (ca. 0.03 μmol m^−2^ s^−1^). Subsequently, the maximal fluorescence (Fm) was measured by applying a saturating actinic light pulse (> 6000 μmol m^−2^ s^−1^) for 0.8 s. Thus, it was possible to calculate the maximum photochemical efficiency of photosystem II (PSII), Fv/Fm = (Fm − Fo)/Fm.

In light-adapted leaf tissues, instant light-response curves were obtained using the light-curve program of the Mini-PAM. The photosynthetic photon flux, ranging from 0 to 1620 µmols m^−2^ s^−1^ for a period of 4 min in eight steps following each other within 30 s. At the end of each light level, a saturating pulse was applied to determine fluorescence parameters. These Chl fluorescence parameters were used to effective photochemical efficiency of PSII (ΦFSII = ΔF/Fm′), the apparent electron transport rate (ETR = (ΔF/Fm′ × PPFD × 0.5 × 0.84), the photochemical quenching (qP = (Fm′-F)/(Fm′ − F0′)) and the non-photochemical quenching [NPQ = (Fm − Fm′)/Fm′; qN = Fm − Fm′/Fv)]^60^. For ETR calculation, it was assumed that quanta were evenly distributed between PSII and PSI (0.5), and leaf light absorption was considered to be 0.84^61^. At 35 days of stress imposition the light curves were presented as delta values (Δ), i.e., the difference between the values of the progenies under water deficit in relation to the irrigated control. This evaluation also included Fv/Fm and electron transport-to-net photosynthesis rate ratio (ETR/A).

### RNA extraction

Samples samples from each replicate stored at − 80 °C were ground into a powder in liquid nitrogen and the total RNA was extracted following the recommendations of the Plant RNA Purification Reagent (PRPR) manual (Invitrogen).

RNA quantification was performed using a NanoDrop 1000 Spectrophotometer (Thermo Scientific, Waltham, MA, USA). RNA integrity was measured by 1% electrophoresis gel analysis of agarose stained with ethidium bromide. The genomic DNA contaminant was removed from the RNA samples by treatment with RQ1 RNase-free DNase (Promega) according to the manufacturer’s instructions.

### qPCR real-time assay

For real-time quantitative PCR, the first strand cDNA was synthesized using 1 μg of total RNA, the ImProm-II Reverse Transcription System and oligo(dT15) according to the manufacturer’s recommendations (Promega, Madison, WI, USA). Real-time quantitative PCR (RT-qPCR) experiments were carried out using the protocol recommended for the use of a 7500 Fast Real-Time PCR System (Applied Biosystems, Foster City, CA, USA) as previously described by Marraccini et al.^21^. Primers (Table 2) were designed using Primer Express software (Applied Biosystems) and were preliminarily tested for their specificity and efficiency against a cDNA mixture from leaves. Data were analysed using 7500 Fast Software v2.0.6 (Applied Biosystems) to determine cycle threshold (Ct) values. The specificity of the PCR products generated for each set of primers was verified by analysing the *T*m (dissociation) of amplified products. PCR efficiency (*E*) was estimated using absolute fluorescence data captured during the exponential phase of amplification of each reaction with the equation (1 + *E*) = 10(− 1/slope)^62^. Expression levels were calculated by applying the formula (1 + *E*^)−ΔΔ*C*t^ where Δ*C*t_target_ = *C*t_targetgene_ − *C*t_*CaUBQ*_ and ΔΔ*C*t = Δ*C*t_target_ − Δ*C*t_reference sample_. Gene expression levels were normalized to the expression level of the *CaUBQ10*^63^ reference gene. Genotype 15 irrigated at zero time was used as internal calibrator with relative expression equal to 1.

**Table 2:** Candidate genes for drought tolerance, protein name, corresponding primers and GenBank (GB) accession numbers of coffee EST sequences used in the qPCR experiment.

Gene	Protein name	Sequence (5′–3′)	GB numbers
*CaDHN1*	Dehydrin	F:CCCCTGGTCTGAGCTCGTT R:GACGCGGAAGTAGGCGTAATT	
*CaEDR2*	EDR1-like MAPKK kinase	F:CGGCATAAGAGCGAGTGGAA R:ATGCAATCGCTGGTGTAGAAAA	DV681462
*CaMYB1*	MYB-type 2 transcription factor	F:CCCGGCAATCTTCCAGCTA R:TCAAGCGTGGCAACTTCACT	GT689406
*CaNCED*	9-cis-epoxycarotenoid dioxygenase 3	F:GCCTGGGAAGAGCCTGAAAC R:CCCCTCGTCACATTCATTGAA	
*CaERF017*	Dehydration-responsive element-binding	F:ATTCCGCCTGGAGCTCAAGT R:GGTGGTCCAGTTGGAGAGTGA	
*CaSDD1*	Subtilisin-like serine protease	F:GAGCCCCGATTGATCTTCTG R:ACTCAGCCCCAAAAGGGTTAA	
*CaGolS3*	Galactinol synthase	F:CCCTTTGGTGGTTGCAGTTT R:AGGCTCGATCTCCCGGACTATA	
*CaAPX1*	Ascorbate peroxidase	F:GACCTGAACAATGCCCAGAAG R:CGTAAATGAGCAGCAGGTGATG	GT697455
*CaAPX6*	Ascorbate peroxidase	F:AGACCGTGTCTCAAACCGACTAC R:GTTGATCTGTTGGCCCAAAGA	EE193467
*CaAPX5*	Ascorbate peroxidase	F:ATCCAGAGGGCAGGGTACCT R:ACCAAAGCCGAGAGCAGTGA	
*CaPYL8a*	Abscisic acid receptor PYL8	F:GGTTTGATCAGCCCCAGAAA R:CCACTTCCCTAAGGCTTCCAA	
*CaPYL8b*	Abscisic acid receptor PYL8	F: GCCAGAGGGAAATACCAAGGA R: CAGCTAGGCGCTCTGAGACA	
*CaPYR1*	Abscisic acid receptor PYR1	F:CGGTGACGACTGTCCATGAG R:TCCGGCACGTCAACGATATA	
*CaAHG3*	Protein phosphatase	F: ACCGGAGGTGACGATAATCG R: CCCACAAGCTGTGTCATTGG	
*CaSNRK 2.8*	Serine/threonine-protein kinase SAPK2	F: CCGCTTCAAAGAGGTCTTGCT R: TTCTCCTCCTGCCGCATACT	
*CaSNRK 2.10*	Serine/threonine-protein kinase SAPK2	F: TCGATTCAAGGAGGTGGTGTT R: TTCCCCTCCAGCTGCATACT	
*CaUBQ10*	Ubiquitin	F: AAGACAGCTTCAACAGAGTACAGCAT R: GGCAGGACCTTGGCTGACTATA	GW488515

### Selection of candidate genes

From previous studies, several candidate genes were identified in different tissues (leaves, roots, plagiotropic buds) of drought-tolerant (DT) and drought-susceptible (DS) plants of *C. canephora*^18–22^ and *C. arabica*^19,23–25^ displaying differential expression profiles under drought stress. Of these genes, 16 candidate genes (Table 2) were selected to be tested on leaves of the four progenies from the crossing ‘Icatu (3851-2-UFV 2117) × Catimor UFV 5373’ under control and drought conditions. These genes corresponded to *CaERF017, CaMYB1, CaEDR2, CaGolS3*, *CaDHN1, CaSDD1, CaAPX1, CaAPX5, CaAPX6, CaNCED, CaPYL8a, CaPYL8b, CaPYR1, CaSNRK2.8*, *CaSNRK2.10* and *CaAHG3.*

## Data Availability

All data generated or analysed during this study are included in this published article.
